# Levels of concentrates and protein sources in intensive pasture-based beef cattle rearing during the rainy season in the Amazon biome

**DOI:** 10.1371/journal.pone.0320629

**Published:** 2025-04-21

**Authors:** Artur Carmanini de Faria, Dheyme Cristina Bolson, Douglas dos Santos Pina, Carla Silva Chaves, Thiago Trento Biserra, Thiago Alves Prado, Dalton Henrique Pereira

**Affiliations:** 1 Grupo de Estudos em Pecuária Integrada - GEPI, Universidade Federal de Mato Grosso, Sinop, Mato Grosso, Brazil; 2 Departamento de Pesquisa e Desenvolvimento da Fortuna Nutrição Animal, Nova Canaã do Norte, Mato Grosso, Brazil; 3 Animal Science Department, Universidade Federal da Bahia, Salvador, Bahia, Brazil; 4 Departamento de Pesquisa da Fundação M.T., Rondonópolis, Mato Grosso, Brazil; University of Agriculture Faisalabad, PAKISTAN

## Abstract

This study aimed to evaluate the effects of different nutritional strategies on the intensification of beef cattle farming on pastures during the rainy season. Eighty male cattle (testers) were randomly allocated to 16 paddocks formed with Mombaça grass (*Megathyrsus maximus*), totaling five animals (testers) per paddock. The strategies consisted of two levels of concentrates (LC) [7 and 10 g.kg^-1^ of body weight (BW)], i.e., low and high LC, and two protein sources (PS) with dried distillers’ grain with solubles (DDGS) and soybean meal (SBM) in a completely randomized design with a 2 × 2 factorial arrangement. The forage’s chemical, structural, and productive characteristics and the supplemented animals’ performance, productivity, and serum parameters were evaluated. The forage had the greatest height (P = 0.038) at the lowest LC; however, a greater leaf proportion (P = 0.049) and leaf: stem ratio (L:S) (P = 0.042) were observed when the highest LC was used. The animals receiving the highest LC had the highest supplement intake (P < 0.001) and a lower pasture intake (P = 0.001). The average daily gain (ADG) did not differ between the LC (P = 0.135) and PS (P = 0.190) groups (0.97 kg.day^-1^). The LC used in the nutritional strategies did not affect the stocking rate (P = 0.272) or productivity (P = 0.986). Supplementation of 7 g.kg^-1^ BW is recommended for intensive rearing of beef cattle during the rainy season, as it results in high gains at a relatively low cost. In addition, as there were no negative responses to the use of DDGS as a protein source in the supplement, it represents an alternative for replacing soybean meal in the formulations.

## Introduction

In Brazil, greater availability and quality of forage are observed in the rainy season, resulting in suitable conditions for beef cattle production on grasslands, allowing a range of grazing intensity strategies. However, despite improvements in animal performance, the use of basal forage during the rainy season is not ideal. According to Detmann et al. [1], tropical pastures experience a nutritional imbalance during this period, characterized by a relative excess of energy compared with the amount of crude protein (CP) available.

In literature, supplementation is a way to improve the nitrogen status of an animal’s metabolism [2], improve the digestibility of nutrients [3], increase dry matter intake [4], and consequently, improve animal performance. Therefore, the use of supplementation, even during the rainy season, is essential for maintaining the growth curve of cattle and reducing the slaughter time of these animals.

Nevertheless, the protein source and the level of concentrates can vary significantly throughout the year, depending on the season and the attributes of the forage, potentially affecting the animals’ response [5,6]. According to a meta-analysis [7], offering energy supplementation at rates >10 g.kg^-1^ body weight (BW) during the rainy season increases gain per area. However, [8] reported greater profitability when cattle received a mineral supplement (3 g.kg^-1^ BW) during the rearing phase, which led to further studies on the levels and quality of supplementation for this phase.

Protein sources are among the costliest components in supplement formulation. Soybean meal is the primary protein source in ruminant diets; however, it is also widely used in other livestock feed, increasing its price. Distillers’ dried grains with solubles (DDGS) offer a viable alternative for beef cattle supplements [9]. DDGS byproducts are also competitively priced, especially in production regions like Mato Grosso, Brazil [10].

Despite the potential gains of animals during the rearing phase, supplementation is not yet a consolidated technique among producers, especially supplementation with high levels of supply prioritized by intensive pasture rearing (IPR) during the rainy season. In addition, the conflict between information on the use of DDGS and the amount of supplements to be offered to animals leaves a gap to be researched. Thus, this study aimed to evaluate the effects of nutritional strategies associated with the levels of concentrate and protein sources on the performance, blood parameters, and forage composition of Nellore cattle during the rearing phase of the rainy season.

## Materials and methods

### Experimental area and ethical approval

The experimental trial took place at the Fortuna Nutrição Animal Company research center (Nova Canaã do Norte, state of Mato Grosso, Brazil:10°24’35” W, 55°43’35” S, altitude 288 m). All procedures adhered to the ethical standards outlined by the Brazilian College of Animal Experimentation (COBEA –Colégio Brasileiro de Experimentação Animal) and received approval from the National Council for Animal Experimentation Control (CONCEA - Conselho Nacional de Controle de Experimentação Animal) of Universidade Federal de Mato Grosso (UFMT), Araguaia Campus (protocol number 23108.042666/2020–94).

**Fig 1:**
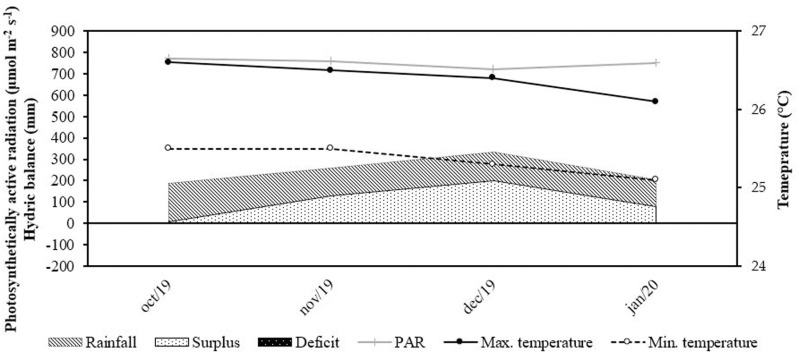
Soil water deficit and excess, accumulated precipitation, minimum and maximum air temperatures from October 2019 to January 2020.

The experimental area consisted of 40 hectares of Mombaça grass pasture (*Megathyrsus maximus*), divided into 16 paddocks covering 2.5 hectares. Each paddock was equipped with a 500-liter drinking trough and a 6-meter linear feed trough, both with free access. According to the Köppen classification, the region has an Am-monsoon climate, with distinct rainy and dry seasons [11]. Climatic data during the experimental period were recorded by a weather station located 500 m from the experimental site (Fig 1). The experimental period, corresponding to the rainy season, lasted from October 2019 to January 2020.

### Animal management and experimental diets

This experiment during the rainy season is a continuation of the study conducted by the same research team and with the same group of animals [5], previously carried out during the dry season. The animals were kept in the same paddocks as those used in the previous experiment. Importantly, the animals that received a high level of concentrate during the dry period (16 g.kg^-1^ BW) were given 10 g.kg^-1^ BW during the rainy period, whereas those that received a low level of concentrate (10 g.kg^-1^ BW) during the dry period were supplemented with 7 g.kg^-1^ BW in the rainy period [5].

Eighty intact Nellore males, averaging 310±10.5 kg, were randomly assigned to paddocks, with 5 animals/paddock. At the start of the experiment, all animals were weighed, handled, treated for internal and external parasites, and randomly allocated to paddocks. Fourteen days later, the animals were weighed again, and at the end of the study, they underwent a 16-hour fasting period from solids and liquids. Additional animals of similar types and weight were introduced to maintain an average forage canopy height of 50 cm [12], using a continuous grazing system with variable stocking rates (heads.ha^-1^). A paddock of the same pasture parallel to the experimental module was intended to receive 90 animals with the same pattern receiving a similar diet to those in the experiment to be used as regulatory animals in grazing management. Importantly, weight gain measurements were carried out solely on the tester animals in all paddocks. These adjustments ensured uniform forage conditions, allowing for the measurement of productivity variables per area within the system.

The nutritional strategies were randomly distributed across each paddock as follows: two levels of concentrate (LC), low and high, at proportions of 7 and 10 g.kg^-1^ of body weight (BW), respectively, using SBM as the protein source (PS); and two levels of LC, low and high, at proportions of 7 and 10 g.kg^-1^ of BW, respectively, using DDGS as the PS. The regulator animals will receive intermediate-level supplementation with DDGS and soybean meal as protein sources.

An adaptation period for supplement intake was unnecessary, as the animals were already accustomed to consuming supplements in amounts greater than those specified in each nutritional strategy. Supplements were produced and supplied by Fortuna Nutrição Animal. The animals received supplements at 2 g.kg^-1^ of BW, introduced gradually to ensure consistent daily protein intake across all animals.

The concentrates were composed of ground corn grain, livestock urea, protected urea (Optigen®, Alltech), a mineral mix, and a vitamin supplement. They were designed to guarantee a consistent protein intake across all treatments, ensuring an isonitrogenous diet (Table 1).

**Table 1:** Ingredient proportion and chemical composition of high (10 g.kg^-1^ BW) and low (7 g.kg^-1^ BW) concentrated supplements based on soybean meal (SBM) and dried distiller’s grains with solubles (DDGS), and Mombasa grass pasture for intensive beef cattle production.

Variables	Levels of concentrates				Pasture
	High		Low		
	SBM	DDGS	SBM	DDGS	
**Proportion of ingredients**					
Ground corn grain	856.18	807.28	692.43	555.64	–
Soybean meal	83.67	–	221.65	–	–
DDGS	–	133.20	–	354.02	–
Protected urea	21.19	21.17	29.94	29.83	–
Common Salt	14.80	14.81	21.05	21.03	–
Limestone	12.77	9.86	20.32	13.04	–
Phoscalcium	4.30	6.32	4.69	12.57	–
BYPRO ^1^	2.48	2.48	3.52	3.51	–
PREMIX MIN. VIT ^2^	4.61	4.89	6.40	10.35	–
**Chemical composition;** *g.kg*^-1^ *of* ***dry matter*** (***DM***)					
DM (g.kg^-1^ as is)	902.6	911.2	933.2	900.6	254.6
pdDM ^3^	958.2	964.9	957.5	957.3	809.66
Ash	46.02	68.18	70.55	69.50	87.75
CP ^4^	172.97	187.72	211.01	239.23	99.20
CF ^5^	22.85	24.51	26.29	43.31	14.72
NDFap ^6^	96.7	93.8	132.7	136.6	721.84
ADF ^7^	56.40	62.75	72.52	103.49	341.38
iNDF ^8^	24.39	17.71	25.97	26.16	204.59
NFC ^9^	694.53	658.82	606.25	544.25	250.82
TDN ^10^	834.11	807.35	768.73	780.81	600.54

^1^Feed supplement containing 70% condensed tannins extracted from red-breasted bream (Schinopsis lorentzii); ^2^ ADV Fortuna, Vitamitract ADE, Fe Sulfate 30%, Mn Sulfate 26%, Chromium Chelate 10%, Sulfur Vent 99%; ^3^ Potentially Digestible Dry Matter; ^4^ Crude Protein; ^5^ Crude fat (petroleum ether extraction); ^6^ Neutral Detergent Fiber corrected for ash and protein; ^7^ Acid Detergent Fiber; ^8^ Indigestible Neutral Detergent Fiber; ^9^ Nonfibrous Carbohydrates; ^10^ Total Digestible Nutrients (Calculated by the NRC [13]).

### Data collection and sampling procedures

Forage collection was conducted every 28 days at ground level, with three samples taken per paddock at the average height of the forage canopy using a circular frame with a known area (0.68 m²). In each paddock, one subsample was used to determine dry matter (DM), while another was separated into morphological components (stem, leaf, and dead material). Simultaneously, a grazing simulation technique was employed to assess the chemical composition of the pasture [14]. Forage accumulation was measured using three exclusion cages per paddock, following the paired-cage method [15]. The average height of the forage canopy was recorded as described by [16]. The volumetric density of the morphological components was calculated by dividing the total dry mass of each component by the average height of the pasture [17]. Grazing pressure was determined by the ratio of animal body weight (BW) (kg) to the amount of forage available (kg BW kg⁻¹·DM·day⁻¹).

The tiller population density, tiller weight, leaf area index, and specific leaf area were assessed every 28 days. The tiller population density (number of tillers.m^-^²) was determined by counting all live tillers within a 0.68 m² metal frame, replicated at six points at the average height of the paddock. Thirty live tillers were sampled close to the ground at the average height of the forage in each paddock. The tillers were weighed, and the leaves close to the stem were removed. The values were then divided by the same number of tillers collected to determine the weight.tiller^-1^ (g.tiller^-1^) and number of live leaves.tiller^-1^ (NLL.tiller^-1^).

To estimate the leaf area index (LAI) of Mombaça grass, the green leaf blades of the collected tillers were used and analyzed with a LI-COR leaf area integrator (model LI 3000). The leaf area was calculated by multiplying the tiller population density (tiller.m^-^²) by the average leaf area of the tiller (m² of leaf area.tiller^-1^), as proposed by [18]. After the leaf blade area of each tiller was measured, the samples were dried in an oven at 55°C for approximately 72 hours, and the dry weight was determined. The leaf area was subsequently divided by the dry weight of the leaf blades of the tiller (cm².g^-1^ DM).

Weekly sampling of each supplement was performed throughout each experimental period for the chemical analysis of the supplement made for each period from a composite sample made from the samples collected weekly.

Chemical composition analyses of the forage and supplement samples were performed at the Laboratory of Nutrição Animal e Forragicultura of UFMT - campus Sinop. The blood analyses were performed at the Núcleo de Pesquisa e Apoio Didático em Saúde (NUPADS), laboratory of Imunopatologia e Doenças Tropicais of UFMT - campus Sinop.

For the chemical analyses of forage and concentrates for DM, ash, and crude protein (CP), the methods of [19] were used. Neutral detergent fiber corrected for ash and protein (NDFap) was quantified by subtracting the ash and protein content from the NDF mass of the sample, following the method described by [20], while indigestible NDF (iNDF, g.kg^-1^) was determined to conform to [21]. Neutral detergent insoluble nitrogen (NDIN, g.kg^-1^) and acid detergent insoluble nitrogen (ADIN, g.kg^-1^) were quantified according to [22], lignin was measured following [23], and total digestible nutrients (TDN, g.kg^-1^) were calculated based on the approach of [13]. Potentially digestible DM (pdDM, g.kg^-1^) was assessed using the technique from [24] and total carbohydrates (TCHO, g.kg^-1^) were analyzed according to [25].

To assess blood parameters, samples were taken from three animals/pen through jugular vein puncture, using sterile 8 mL Vacuette® tubes (Kremsmünster, Austria) containing a coagulation accelerator. The samples were centrifuged at 3,000 × g for 15 minutes to separate plasma and serum. The serum was then stored at -20°C for later analysis of urea, creatinine, uric acid, alkaline phosphatase, total proteins, albumin, bilirubin, aspartate aminotransferase (GOT/AST), alanine aminotransferase (GPT/ALT), and gamma-glutamyltransferase (GGT) contents, using an enzymatic-colorimetric method with commercial kits (Gold Analisa®, Belo Horizonte/MG, Brazil).

### Performance and feed intake measurements

Performance evaluations were based on the weight of tester animals after a 16-hour fast, while productivity metrics were calculated using data from all animals managed within each paddock. The metrics were calculated as follows: Average daily gain (ADG, kg BW.day^-1^), Feed efficiency (FE, BW gain (kg)/total intake(kg), dmls), Stocking rate (SR, 450 kg.BW.ha^-1^; Eq. (1)), Total weight per area (TWG, kg.ha^-1^; Eq. (2)), Gain weight per area per day (GAD, (kg.(ha×day)^-1^)); Eq. (3), and Productivity (TWG/30, @.ha^-1^).


$$\text{SR}=\frac{\left(\sum \text{BW}/450\right)}{\text{area }}$$ (1)

$$TWG=\frac{\sum BWgain}{area}$$ (2)

$$GAD=\frac{\left(BWgain/area\right)}{days}$$ (3)

To estimate the actual intake of supplements (kg.day^-1^), the quantities supplied daily at 7 a.m. were measured per paddock, disregarding leftovers when they exist. Measurements of supplement disappearance were performed 3, 6, 9, 12, 15, 18, 21, and 24 hours after supply. To estimate the intake of total dry matter in the diet (total DMI), the following equation was used [26]:


$$TotalDMI=-1.7824+\left(0.07765\times BW^{0.75}\right)+\left(4.0415\times ADG\right)-0.8973\times ADG^{2}$$ (4)

### Statistical analysis

The analyses followed a completely randomized design, using a 2×2 factorial arrangement (two protein sources and two concentrated supplementation levels), resulting in four treatments with four replicates (paddocks) per treatment. The model applied was:


$${\text{Y}}_{ijk}=\text{μ}+\alpha_{i}+{\text{β}}_{j}+{\left(\alpha \beta \right)}_{ij}+\delta \left(Y_{ijk}\right)+e_{ijk}$$ (5)

which ${\text{Y}}_{ijk}$ is the observation of the treatment effect, μ overall mean, $\alpha_{i}$ is the effect of concentrate level, ${\text{β}}_{j}$ is the effect of protein source, ${\left(\alpha \beta \right)}_{ij}$ represents the interaction between concentrate level and protein source, $\delta \left(Y_{ijk}\right)$ is the initial body weight used as a covariate, and $e_{ijk}$ denotes the residual errors associated with each observation.

The individual performance (ADGs) and serum parameters were analyzed using the model proposed by [27]:


$$Y_{ijk}=\text{μ}+\alpha_{i}+\beta_{j}+{\left(\alpha \beta \right)}_{ij}+a_{l\left(k\right)}+{\text{e}}_{ijk}$$ (6)

where $Y_{ijk}$ represents the measurement for the animal in paddock *k* within repetition *j*; μ is the overall mean; $\alpha_{i}$ denotes the fixed effect of protein source; $\beta_{j}$ represents the fixed effect of concentrate level (*j* =7 and 10 g.kg^-1^ BW); ${\left(\alpha \beta \right)}_{ij}$ is the interaction effect between protein source and concentrate level; $a_{l\left(k\right)}$ is the random sampling error for each animal (*l*) within paddock (*k*); and ${\text{e}}_{ijk}$ is the random experimental error for each observation. The F-test was applied in all evaluations with a 0.05 significance level for type I error.

## Results

### Forage characteristics

The variables related to the tiller population were not affected (P > 0.05) by the LC*PS interaction and did not affect (P > 0.05) the LC or PS (Table 2).

**Table 2:** Assessment of the tiller population of Mombaça grass (*Megathyrsus maximus*) under continuous stocking during the rainy season.

Variables	LC ^4^		PS ^5^		SEM ^1^	P value		
	Low	High	DDGS ^6^	SBM ^7^		LC	PS	LC*PS
Height (cm)	54.65	50.85	52.31	53.19	1.55	0.06	0.63	0.53
Number of leaves	5.35	5.15	5.23	5.26	0.11	0.23	0.86	0.38
Number of tillers	428.86	423.19	433.16	488.89	10.31	0.71	0.35	0.64
LAI ^2^	4.80	4.93	4.87	4.86	0.23	0.72	0.96	0.63
Leaf area	113.07	116.47	113.04	116.49	5.04	0.64	0.64	0.42
Weight tiller (g.tiller^-1^)	3.34	3.13	3.28	3.19	0.14	0.31	0.67	0.38
LAI ^3^ specific	108.08	110.45	108.81	109.72	3.10	0.83	0.57	0.29

^1^Standard error of the mean; ^2^ Leaf area index; ^3^ Specific leaf area index; ^4^ Level of Concentrate: Low (7 g.kg^-1^ DM) and high (10 g.kg^-1^ DM); ^5^ Protein sources; ^6^ Dried distillers’ grain with solubles; ^7^ soybean meal.

The height of Mombaça grass was affected (P = 0.038) by the LC offered, with greater heights in the lowest LC. The forage mass (FM) did not differ between LC and PS (P > 0.05), with a mean value of 5081.11 ± 241.27 kg of DM.ha^-1^. Compared with the lowest LC, the highest LC was associated with an increase of 12.6% in the proportion of leaves (P = 0.049). However, the proportion of stems (P = 0.315) and the proportion of dead material (P = 0.378) were not affected by LC (Table 3).

**Table 3:** Characteristics of Mombaça grass (*Megathyrsus maximus*) under continuous stocking during the rainy season.

Variables	LC ^9^		PS ^10^		SEM ^1^	P value		
	Low	High	DDGS^11^	SBM^12^		LC	PS	LC*PS
Canopy height (cm)	56.06a	51.14b	53.98	53.23	1.43	0.04	0.72	0.84
FM² (kg DM.ha^-1^)	5205.75	4956.47	5322.38	4839.84	172.70	0.33	0.08	0.32
Leaf proportion (g.100 g^-1^)	39.72b	45.48a	41.54	43.66	1.84	0.05	0.43	0.25
Stem proportion (g.100 g^-1^)	27,27	24.86	25.49	26.63	1.60	0.31	0.62	0.32
Dead material proportion (g.100 g^-1^)	33.01	29.66	32.96	29.71	2.62	0.38	0.39	0.16
L: S³	1.78b	2.22a	0.198	2.02	0.13	0.04	0.82	0.75
Leaf VD ^4^ (kg of DM.ha^-1^.cm^-3^)	38.2b	44.13a	41.31	41.01	2.01	0.04	0.91	0.95
Stem VD ^4^ (kg of DM.ha^-1^.cm^-3^)	25.5	24.03	24.6	24.94	1.11	0.38	0.83	0.73
Total VD ^4^ (kg of DM.ha^-1^.cm^-3^)	95.58	98.06	99.98	93.66	4.71	0.71	0.37	0.49
FA ^5^ (kg DM.ha^-1^.day^-1^)	96.36	100.73	98.61	98.48	2.61	0.27	0.97	0.89
Grazing pressure	0.37	0.35	0.35	0.37	0.03	0.62	0.66	0.56
**Chemical composition**								
Dry matter (g.100 g^-1^)	25.34	25.57	25.63	25.28	0.41	0.70	0.57	0.09
pdDM ^6^ (g.100 g^-1^)	79.01	79.25	79.31	79.04	3.36	0.75	0.58	0.86
Organic matter (g.kg^-1^)	912.48	912.01	910.70	913.79	1.05	0.76	0.06	0.24
Crude protein (g.kg^-1^)	101.69	104.24	101.43	104.50	1.46	0.25	0.17	0.22
NDFap ^7^ (g.kg^-1^)	636.09	634.44	634.63	635.91	2.39	0.64	0.71	0.51
iNDF ^8^ (g.kg^-1^)	205.22	203.96	205.36	203.82	3.35	0.74	0.57	0.84

¹ standard error of the mean. ²forage mass; ^3^ Leaf:stem ratio; ^4^ Volumetric densities; ^5^ Forage accumulation; ^6^ potentially digestible dry matter; ^7^ Neutral detergent fiber corrected for ash and protein; ^8^ Indigestible neutral detergent fiber; ^9^ Level of Concentrate: Low (7 g.kg^-1^ DM) and High (10 g.kg^-1^ DM); ^10^ Protein sources, ^11^ Dried distilled grains with solubles; ^12^ Soybean meal. Means followed by the same letters in the line do not vary among themselves by the F test considering a 5% probability for type I error.

The LC affected the L:S ratio (P = 0.042) and leaf volumetric density (VD) (P = 0.041). The L:S ratio at the highest LC was 20% greater, and the leaf VD was 13% greater than that at the lowest LC (Table 3).

For stem VD (P=0.376), total VD (P=0.715), and forage allowance (FA) (P=0.267), there were no differences between the LCs, with averages of 24.76 ± 0.73 kg of DM.ha^-1^.cm^-1^ and 96.82 ± 3.16 kg of DM, respectively. ha^-1^.cm^-1^ and 99.24 ± 1.49 kg of DM. ha^-1^.day^-1^, respectively (Table 3).

The chemical composition of the forage did not affect (P > 0.05) the LC*PS interaction and did not differ for LC (P>0.05) or PS (P>0.05) for all the variables evaluated (Table 3).

### Animal consumption and performance

There was no interaction effect on LC*PS (P > 0.05) or effect (P > 0.05) on LC or PS for any of the productive performance variables measured (Table 4). The average initial body weight (IBW) was 320 kg, and the average final body weight (FBW) was 393.14 kg. The ADG was 0.97 kg.day^-1^. Similarly, the average feed efficiency was 122.42 g.kg^-1^ DM supplement. Furthermore, TWG and carcass weight gain (CWG) had average values of 475.98 ± 84.62 kg.ha^-1^ and 6.3 ± 1.07 kg.ha^-1^.day ^-1^, respectively.

The SR (P=0.272) and productivity (P=0.428) were not affected by the concentration, with averages of 4.74 ± 0.57 AU.ha^-1^ and 15.86 ± 2.82@.ha^-1^, respectively.

The supplement DMI (sDMI) (P<.001) and pasture DMI (pDMI) (P<.001) differed for LC; the animals that were subjected to the highest LC had a supplement consumption 30% higher, whereas the pasture consumption was 16% lower, concerning the animals that were in the lowest LC (Table 4).

**Table 4:** Performance of beef cattle reared on Mombaça grass pasture (*Megathyrsus maximus*) supplemented with high levels of concentrate and different protein sources during the rainy season.

Variables	LC ^10^		PS ^11^		SEM¹	P value		
	Low	High	DDGS ^12^	SBM ^13^		LC	PS	LC*PS
Initial body weight (kg)	315.93	324.07	321.10	318.90	5.21	0.30	0.77	0.57
Final body weight (kg)	388.96	392.61	385.40	395.60	5.18	0.60	0.20	0.90
Supplement intake (%BW.day^-1^)	0.61b	0.87a	0.75	0.73	0.01	<.001	0.19	0.83
Average daily gain (kg.day^-1^)	0.92	1.02	0.92	1.01	0.05	0.14	0.19	0.45
Feed efficiency	12.02	12.46	12.06	12.42	0.22	0.18	0.26	0.30
Total body weight gain	493.87	458.10	455.28	496.70	28.92	0.43	0.34	0.27
Area body weight gain	6.53	6.06	6.02	6.57	0.36	0.41	0.31	0.42
Stocking rate (AU. ha^-1^)	4.92	4.55	4.75	4.72	0.21	0.27	0.92	0.39
Productivity (@.h ^-1^)	16.46	15.27	15,17	16.55	0.99	0.43	0.34	0.27
**Intake** ^2^								
Total DMI ^3^ (kg of DM.day^-1^)	7.41	7.66	7.44	7.62	0.13	0.17	0.34	0.53
DMI %BW ^4^	2.10	2.12	2.12	2.10	0.02	0.47	0.47	0.42
sDMI ^5^ (kg of DM.day^-1^)	2.35b	3.44a	2.91	2.88	0.02	<,001	0.15	0.32
pDMI ^6^ (kg of DM.day^-1^)	5.05a	4.21b	4.53	4.73	0.13	<,001	0.25	0.60
CPI ^7^ (kg of DM.day^-1^)	1.04	1.06	1.06	1.04	0.01	0.51	0.21	0.63
NDFI ^8^ (kg of DM.day^-1^)	3.62a	3.12b	3.30	3.43	0.08	<,001	0.26	0.68
NFCI ^9^ (kg of DM.day^-1^)	2.34b	3.18a	2.70b	2.82a	0.03	<,001	0.01	0.24

¹Standard error of the mean; ^2^ Intake was calculated via equation 2.1, BR Corte 5.0 [27]; ^3^ Total dry matter intake; ^4^ Dry matter intake as a percentage of body weight; ^5^ Supplement dry matter intake; ^6^ Pasture dry matter intake; ^7^ Crude protein intake; ^8^ Neutral detergent fiber intake; ^9^ Nonfibrous carbohydrate intake. ^10^ Concentrations: low (7 g.kg ^-1^ DM) and high (10 g.kg ^-1^ DM); ^11^ protein sources. Means followed by the same letters on the line do not differ from each other according to the F test considering a 5% probability for type I error.

The crude protein intake (CPI) did not differ between LCs (P=0.509), with an average of 1.05 kg.day^-1^. The neural detergent fiber intake (NDFI) and nonfibrous carbohydrate intake (NFCI) differed between LCs (P <0.001), with greater NDF intake observed in the lowest LC and greater NFC intake in the highest NS. The NFCI also had an effect (P=0.002) on PS, which was greater when SBM was used as the PS (Table 4).

**Fig 2:**
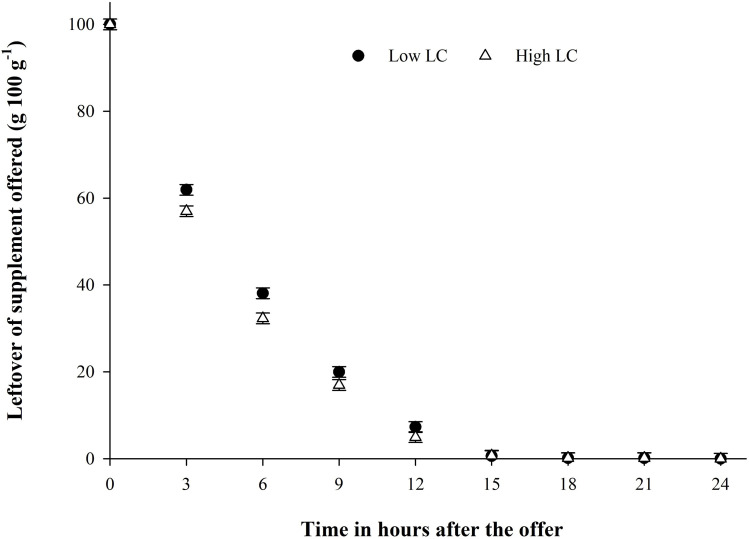
Segmented linear regression model for supplement levels of 7.0 (P<0.0001) and 10.0 (P<0.0001) g.kg^-1^ of body weight for the relationship between leftovers and time after supplement offering: Ŷ7.0= 94.58–8.80*time for time ≤ 10.11 hours and Ŷ7.0 = 0.00 for time ≥ 10.11 hours (R2 = 98.38) and Ŷ10.0 = 92.63–9.13*time for time ≤ 9.70 hours and Ŷ10. 0 = 0.00 for times ≥ 9.70 hours (R2 = 97.19).

**Fig 3:**
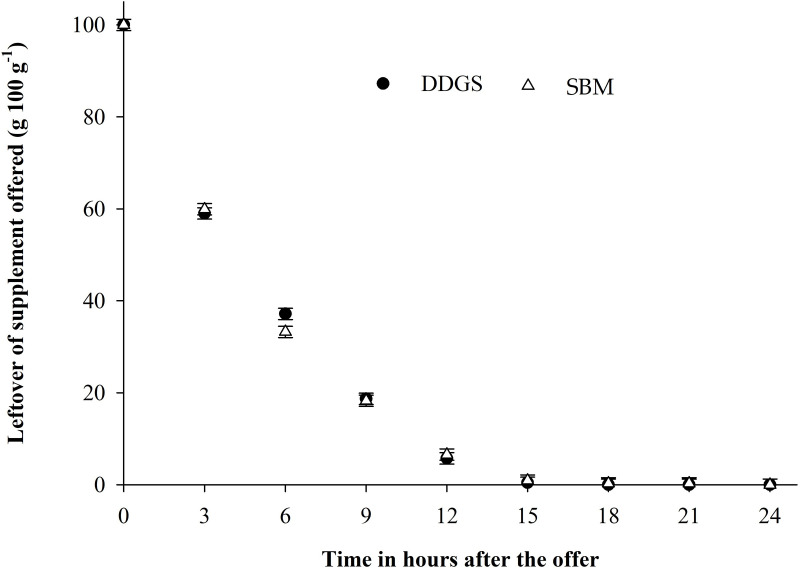
Segmented linear regression model for the protein sources DDG´S (P<0.0001) and SBM (P<0.0001) for the relationship between leftovers and time after supplement offering: ŶDDG´S= 93.58–8.86*Time for Time ≤ 10.06 hours and ŶDDG´S = 0.00 for Time ≥ 10.06 hours (R2 = 97.96) and ŶSBM= 93.63–9.06*Time for Time ≤ 9.74 hours and ŶSBM= 0.00 for Time ≥ 9.74 hours (R2 = 97.67).

The disappearance of the supplements throughout the experimental period is shown in Figs. 2 and 3. All the supplements offered were consumed within 12 hours after supply, regardless of the LC evaluated. The lowest LC presented a relatively high disappearance rate, averaging 9.12 g.100 g^-1^, up to 9 hours after supply, but reached 12 hours after supply, with all the supplements offered consumed by the animals.

When the effect of PS on the disappearance of the supplement was evaluated, it was observed that all of the supplement offered was consumed within 12 hours of being supplied (regardless of PS) (Fig. 3).

### Blood parameters

In the evaluation of the serum profile of the animals (Table 5), there was no interaction effect between LC*PS (P>0.005) and any of the parameters evaluated. LC evaluated under the different nutritional strategies did not influence (P>0.005) the serum profile of the animals.

PS influenced (P=0.049) the concentration of total proteins in the animals, with a higher concentration (75.57 g.dL^-1^) when the animals received DDGS as the PS. However, when the serum concentration of urea was evaluated, the highest concentration (P=0.012) occurred in the animals that received SBM as PS (Table 5).

**Table 5:** Serum profiles of beef cattle receiving different nutritional strategies as a strategy to intensify the rearing phase during the rainy season of the year.

Variables	LC ^5^		PS ^6^		SEM¹	P value		
	Low	High	DDGS ^7^	SBM ^8^		LC	PS	LC*PS
Cholesterol (mg.dL^-1^)	144.53	136.29	139.32	14149	3.64	0.35	0.80	0.73
Uric Acid (mg.dL^-1^)	1.87	1.32	1.60	1.59	0.49	0.44	0.98	0.77
Albumin (g.L^-1^)	32.22	30.87	32.16	30.93	1.04	0.38	0.41	0.52
Total Proteins (g.L^-1^)	69.54	73.80	75.57a	67.77b	2.84	0.27	0.05	0.18
GOT ^2^ (U.L^-1^)	117.99	127.09	121.29	123.79	6.09	0.30	0.77	0.28
GPT ^3^ (U.L^-1^)	75.13	72.96	77.71	70.37	5.03	0.75	0.29	0.20
Alkaline Phosphatase (U.L^-1^)	100.27	112.94	98.36	114.86	20.24	0.66	0.55	0.32
Urea (mg.dL^-1^)	30.12	26.57	24.39b	32.3a	2.08	0.30	0.01	0.16
GGT ^4^ (U.L^-1^)	52.35	46.25	44.73	53.87	3.99	0.28	0.10	0.18

¹ standard error of the mean; ^2^ Glutamic-oxaloacetic transaminases; ^3^ Glutamic-pyruvic transaminase; ^4^ Gamma glutamyl transferase; ^5^ Concentrations: low (7 g.kg^-1^ DM) and high (10 g.kg^-1^ DM); ^6^ Protein sources; ^7^ Dried distillers’ grain with solubles; ^8^ Soybean meals. Means followed by the same letters in the line do not differ from each other according to the F test considering a 5% probability for type I error.

## Discussion

Few studies in the literature have investigated the characteristics of forage as a result of the levels or types of concentrates used [4,28,29]. However, [29] reported that different types of supplements (mineral salt, protein, and/or protein energy) did not alter the characteristics of Tanzania grass (*Megathyrsus maximus*) for beef cattle across various times of the year. In the present study, characteristics such as the number of live leaves, leaf area index (LAI), and leaf area per tiller were consistent with values reported in the literature for Mombaça grass[12,30,31]. This finding demonstrated that the intensification of rearing can be carried out in pasture areas without causing morphogenic changes in the grass.

The LAI is the result of the leaf appearance rate (LAR), leaf elongation rate (LER), and leaf lifespan (LLS); similarly, both characteristics are determined from the plant phyllochron [32]. Thus, we can assume that the maintenance of the LAI in the mombaça grass pasture among the evaluated factors is due to the low capacity of these factors to interfere with the forage phyllochron since the environment (temperature, radiation) and pasture management (without nutrient replacement and tiller height) were similar across all the experimental units.

The lower forage canopy height observed with the highest LC is probably due to pasture management during the dry season, which extended into the rainy season. Animals with higher LCs were heavier, as verified in [5], and remained on the same pastures upon entering the rainy season. Thus, the high stocking rate in the dry season resulted in a lower forage canopy, allowing greater light entry, which ensured a greater proportion of leaves, greater volumetric leaf density, and a higher L:S ratio in the highest LC. For this reason, even though there was no significant difference in FM, the pasture where the animals received higher LC had lower FM than the pasture where the animals received lower LC.

Another relevant aspect is that grazing behavior and forage intake can be influenced by grazing height and supplementation [33,34]. Grazing cattle tends to be selective, preferring plant fractions with relatively high nutritional value, such as green leaves. However, despite the better forage canopy structure (i.e., a greater proportion of leaves), when animals received higher LC, there was a reduction in pasture intake and an increase in supplement intake. As indicated by [35], forage can be replaced with supplements. Thus, in pasture-based animal production systems, higher concentrate intake can increase production costs. However, it can be explored as a management strategy, especially in intensive pasture-based rearing or finishing, where supplementation would promote increases in DMI and consequently in animal performance.

On the other hand, variations in the roughage/concentrate ratio should be considered, especially when higher proportions of concentrate are suddenly introduced, which may disturb rumen conditions [36]. Our results show that LC did not alter the total DMI but rather both pasture and concentrate intake. However, although the concentrate ratio increased, the roughage/concentrate ratios for cattle intensively raised on pasture were 55/45 and 68/32 for relatively high and low LCs, respectively, which is considered appropriate for efficient rumen function [37].

Concerning forage quality, even with a greater proportion of leaves in the pasture when the animals received higher LC, there was no significant difference in the nutritional value of the forage, which was also not influenced by PSs. Forage quality can vary depending on pasture management, fertilization, the physical and chemical characteristics of the soil, and climatic conditions, among other factors [6]. In the present study, all pastures, regardless of whether they were LC or PS, were managed at the same height and did not receive any type of fertilization or soil correction, which may have contributed to the lack of difference in the nutritional value of Mombaça grass between the LC and PS.

Supplement disappearance across nutritional strategies followed a similar pattern, with nearly all supplements consumed within 12 hours of provision. Due to the high intake and substantial inclusion of corn, conventional methods for controlling consumption, such as adding salt or urea, proved ineffective, leaving physiological control as the only way to regulate supplement intake. The peak intake occurred within the first three hours after feeding, particularly with higher levels of LC, as the animals preferred to graze during the cooler parts of the day-either in the morning or late afternoon [38].

There was no difference in the ADG between the LC and PS groups. According to Benedeti et al. [39], the CP requirement for zebu cattle on pasture, aiming at a gain of 0.8 kg BW.day^-1^, is 744 g CP.day^-1^. Even if the supplement intake provided 530 and 620 g of CP.day^-1^, for the lowest and highest LCs, respectively, the high concentration of protein in the forage was sufficient to meet the protein needs of the animals, even exceeding them. This may have contributed to the absence of differences in the ADG of the animals. Moreover, during the rainy season, the animals consumed enough CP to meet their requirements and ensure adequate ruminal fibrolytic activity, as the protein concentration exceeded 7 g.kg^-1^ [40]. Concentrations below this level impair microbial growth, creating suboptimal conditions [41].

Soybean meal represents one of the main sources of protein in diets due to its nutritional profile and is present mainly in ruminant diets, as it is a source of rumen degradable protein (RDP). The lack of effect of PS on any of the performance variables evaluated demonstrates that the use of DDGS represents an alternative for feeding ruminants [42]. DDGS did not alter animal performance in pasture or confinement, serving as a viable alternative to replace conventional ingredients in both systems in a tropical environment.

The average serum levels of total proteins observed for animals raised on pasture receiving different levels of concentrates combined with protein sources are within the parameters considered normal, that is, 73 g.L^-1^ [43]. However, it was observed that animals fed DDGS had higher total protein levels in the blood. The increase in the total protein content in the blood serum of the animals indicates better protein digestibility and proteins of great value for the microorganisms that inhabit the rumen [44]. In addition, the use of supplements with sources of non-degradable protein in the rumen (NDPR), such as DDGS, favors greater absorption of intestinal protein in animals, especially in animals in the rearing phase, which would justify the greater retention of nitrogen by these animals.

Blood urea levels can be used as an indicator of protein status in cattle [45]. Urea values within the optimal range (<64.86 mg.dL ^-1^) in cattle indicate that effective ruminal degradable protein (ERDP) is adequate [46]. In the present study, the protein sources differed in terms of blood urea content, with higher values for animals fed soybean meal. [47] reported that the protein fractions of soybean meal are more degradable than those of DDGS, thus causing an increase in blood urea levels because of protein degradation. However, [48] suggested that blood urea values greater than 5–8 mg.dL^-1^ were indicative of excessive N intake and N wastage. Furthermore, [49] reported that for growing steers, blood urea levels between 11.1 and 15.2 mg.dL^-1^ were associated with maximum rates of gain. These values suggest that, for the present study, the CP content in the diets may have been above the minimum concentration required for the animals.

The enzymes GPT and GOT are indicators of stress and affect blood components. The increased activity of these enzymes is related to the physiological state and is also a phenomenon that accompanies metabolic disorders [50]. According to Kaneko [50], the ideal levels for GOT, GPT, and GGT for cattle are 78–132 U.L^-1^, 11–40 U.L^-1^, and 6.1–17.4 U.L^-1^, respectively. The levels of GPT and GGT found in the present study are above those expected for cattle, and according to Kaneko [50], increases in serum GPT and GGT activity are specific for hepatocellular and pancreas/kidney injury, respectively.

## Conclusions

The supplementation of 7 g.kg^-1^ BW is recommended for intensive beef cattle rearing during the rainy season, as it provides high gains at a lower cost. Furthermore, as there are no negative responses to the use of DDGS as a protein source in the supplement, it represents an alternative for replacing soybean meal in formulations.
